# Venereal Disease

**Published:** 1920-04-10

**Authors:** 


### Venereal Disease.

### Early Treatment Experiment in Manchester.

A most useful report on the first three months' work of the Venereal Diseases Early Treatment Centre for Males, in Great Bridgewater Street, Manchester, has been prepared by Dr. Young, assistant to the Medical Officer of Health. During that period there were 1,016 attendances, 708 persons received treatment, and 117 were referred to treatment clinics in the city. At first treatment was given to all persons who had been exposed to infection within a day or two of appearing at the centre, though they were at the same time informed that the treatment could not be relied upon under such conditions. But since January 5 no treatment has been given in cases where the interval between exposure and treatment was twenty-four hours or over. Only one such case has been dealt with since that date, and he was referred to a treatment clinic. It is now intended to limit the interval to twelve hours.

The experiment has proved (says the report) that men will not only come for early treatment, but will come within a reasonably short time after exposure to infection; that with the exception of two instances there have been no habitual frequenters; and that the centre is evidently a useful means of preventing the spread of venereal disease. The report recommends that, with the approval of the Ministry of Health, the centre should be allowed to continue in operation, and that another centre should be opened at the Victoria Street public convenience. The cost in Great Bridgewater Street has amounted to 2s. 0½d. per head, and 75 per cent. of the cost will be paid by the State.

